# Marginal adaptation and fracture resistance of recent 3D printed high-performance polymer and CAD/CAM hybrid ceramic endocrown restorations and designs

**DOI:** 10.1186/s12903-026-08095-y

**Published:** 2026-04-16

**Authors:** Mohamed Atif Elkholy, Amira Mohammad Samy Mostafa, Hoda Gaafar Hassan Hammad

**Affiliations:** 1 Prosthodontic Department, Faculty of Dentistry, Menoufia University, Shebin Elkom, Egypt; 2 Conservative Dentistry Department, Badr University in Cairo, Cairo Egypt. Affiliated to Zarqa University, Zarqa, Jordan; 3 Prosthodontic Department, Faculty of Dentistry, Menoufia University, Shebin Elkom, Egypt

**Keywords:** Nanoksa G-PLUS, VITA ENAMIC, Nano-zircon, Nano-carbon, Hybrid Ceramic, 3D Printed, CAD/CAM, Endocrown, Anatomic Preparation Design, Butt Joint Preparation Design, Marginal gap distance, Marginal Adaptation, Fracture Resistance

## Abstract

**Aim (Background):**

The purpose of the current in vitro study was to evaluate and compare the marginal adaptation and fracture resistance of CAD/CAM VITA ENAMIC hybrid ceramic and, 3D printed Nanoksa G-PLUS high-performance polymer endocrown composite restorations constructed for endodontically treated teeth *“EET”*; considering two distinct coronal preparation designs: standardized anatomic and butt joint.

**Materials and methods:**

A total of 40 freshly extracted intact human permanent mandibular molars were endodontically treated to receive endocrowns. Specimens were randomly divided into two equal groups “n = 20 each”: Group1 “G1” received 3D printed Nanoksa G-PLUS high-performance polymer, and Group2 “G2” received CAD/CAM VITA ENAMIC hybrid ceramic endocrown restorations. Each main group was further subdivided into two equal subgroups “n = 10 each” based on the coronal preparation design: standardized anatomic “G1A, G2A” and butt joint “G1B, G2B”. All endocrowns were adhesively luted with their respective manufacturer-recommended resin cements. Subsequently, all specimens underwent simulated aging through a chewing simulator and thermo-cycling (CSTC device, CS-4, SD Mechatronik, Germany). Marginal adaptation was quantitatively assessed by measuring the marginal gap distance using a USB Digital stereomicroscope (U500x Digital Microscope, Guangdong, China). Fracture resistance was determined using a universal testing machine (Model 3345; Instron Industrial Products, Norwood, MA, USA). Statistical analysis was performed using IBM SPSS Statistics (version 26), employing a two-way ANOVA followed by Tukey’s post-hoc tests, with significance set at *P* < 0.05.

**Results:**

VITA ENAMIC specimens exhibited significant higher fracture resistance values; (*p* < 0.0001) and significant lower marginal adaptation (higher marginal gap values) than those of Nanoksa G-PLUS.

**Conclusions:**

Successful serviceability of recent endocrown restorations “Nanoksa G-PLUS and VITA ENAMIC” seems critically influenced by the material of construction and the technology of its fabrication. VITA ENAMIC endocrowns combined with anatomic preparation designs yielded the highest fracture resistance, while the Nanoksa G-PLUS endocrowns with butt joint preparation design exhibited the least fracture resistance of all subgroups.

**Clinical significance:**

Understanding the performance characteristics of these advanced technology of restorative biomaterials, and adhesive bonding systems and preparation designs is crucial for optimizing the clinical outcomes and enhancing serviceability and longevity of endocrown restorations in posterior teeth.

## Introduction

After root canal therapy, the tooth is characterized by: obviously reduced nociception, loss of inherent pulp and marginal ridges’ structural integrity, and aged dentin because of the irrigating solutions used during cleaning and shaping of the root canal system [1]. Consequently; the reported biomechanical properties of endodontically treated teeth “*ETT*” were different from those of intact vital teeth [2]. However, the endodontic treatment was properly achieved, the predominant frequent cause for extraction of *ETT* is failure of the coronal portion [3]. In addition, posterior *ETT* are more prone to a greater risk of tooth fracture because of exposure to high masticatory occlusal stresses that may compromise retention of coronal restoration [4].

Evidently, proper decision for restoration of root filled teeth “*RFT*” presents a paramount challenge for dentists because the complications and failure may lead to *“Split-Tooth Syndrome”*, root factures, and consequently, complete teeth loss [5]. Interest in *“Minimally Invasive Endodontic- Restorative Concepts "* represents an important evolution in conservative dentistry that targets preserving the precious architecture of enamel and dentine hydroxyapatite [6].

Fracture resistance of *ETT* is directly related to the remaining hard tooth structure. Therefore, restoration of severe RFT tissue loss was usually performed using a type of post and core system; aiming to provide the necessary resistance and retention forms prior to placement of the permanent coronal restoration [7]. In general, intra-canal metal posts had reported greater fracture resistance compared to those of other post materials [5]. However, a potential danger when using those metal posts had increased risk of non-restorable tooth or root failures [5, 7]. Compared to metal posts, the fabricated reinforced fiber-posts combined with resin composite cores to restore *RFT* clinically showed significant lesser failure rates; which might be due to favorable stress distributions, biomechanical properties, and fracture resistances [7, 8]. Moreover, proponents for *“Minimally Invasive-Endodontic Techniques*” declared that the removal of the peri- cervical dentine, associated with traditional procedures of coronal access cavity and root canal cleaning and shaping might predispose the residual hard tooth structure to dramatic crown and/or root fracture [9–11].

Accordingly, the inquiry for partial coverage composite and ceramic restorations associated with the technology of nano-adhesive systems is rapidly developing to support and preserve the remaining *ETT* structure for esthetic and function [6, 7]. Previously, the gold standard for restoration of *RFT* with extensive loss of hard tooth structure was fabrication of full coverage ceramo-metal or all ceramic crowns that might be used with or without intra-radicular posts [11, 12]. Over the years, as preservation of the precious tooth tissue “Conservatism Concept” dominated, along with the awesome evolution of dental biomaterials, the traditional aggressive macro-mechanical approaches were abolished and replaced by the introduced “*Minimal Invasive Principles of Adhesive Dentistry*” [12, 13]. Therefore, in 1995, *Pissis* had first introduced the endocrown concept that was known as “Monobloc Porcelain Technique”; as an alternative treatment modality that use the *RFT* pulp chamber space and the adhesive systems to increase the macro and micro-mechanical coronal retention [14]. In 1999, Bindl and Mormann were first to coin the *“Endo-crown”* term [15].

Afterword, innovation and rapid development of computer-aided design/computer-aided manufacturing (*CAD/CAM*) technology have opened new spacious horizons, both in biomaterial processing, as well as in restoration of *RFT*, providing excellent biocompatibility, amazing aesthetics, high accuracy and marginal fit, good micro-mechanical retention and less time consumption for the minimally invasive adhesive restorative procedure [14, 16]. Concept of minimally invasive tooth design preparation and achievement of maximum hard tissue conservation are successful using endocrown as a *single mono-block* restoration. Endocrown is suitable for rehabilitation of *ETT* that is severely damaged and/or reduced inter-occlusal space when the space is not enough for an all ceramic restoration or a ceramo-metal substructure. It contains the entire coronal and intra-radicular tooth portions [16–18]. Nowadays, the biomimetic restorative approach has guided development of advanced hybrid *CAD/CAM* ceramic biomaterials that are tailored for the clinical context, by milling into thin layers. Endocrowns fabricated using *CAD/CAM* technology have demonstrated superior surface textures and anatomic contours. Therefore, leveraging the superior nano-zirconia endocrown properties seems an optimal viable conservative solution for restoring *RFT* with improved restoration longevity and obviously reduced risk of failure [19–21].

Worldwise; the awesome progress in nanotechnology and digital dentistry lead to revolution of Nanoksa G-PLUS that presents magic comprehensive solutions for permanent restorations constructed over dental implants including crown, fixed bridge, or complete denture. Nanoksa G-Plus is a commercially available blend of biocompatible high-performance methacrylate’s polymers containing Nano-zirconia and Nano-carbon. The presentation of manufacturer’s Nanoksa G-Plus is in the form of a resin or a disc; where the resin is indicated for fabrication of inlays, onlays, laminate veneers, and single crowns. According to the manufacturer; Nanoksa G-Plus disc when immersed in water at 23 °C for 24 h, it showed absorption of minute amount of water of only 0.1%. Moreover, Nanoksa G-Plus as a highly crystalline structure was insoluble in the ordinary solvents at ambient air temperature. According to International Organization for Standardization (ISO) 527:2019 and ISO 178:2019, the material reported 90–100 MPa as yield strength, and in tensile tests 95–110 MPa as the breaking load. In addition; the Nanoksa G-Plus disc is designed to function with a range of restorative materials, like: composites, metals, and ceramics [18].

The main advantage of this material is its unique composition that combines stress absorption and flexibility. Nanoksa G-PLUS is constructed of an in-tended unique characteristic combination of biopolymers to withstand breakage and occlusal wear while offering a smooth consistent cut and excellent abrasion resistance. According to manufacturers, Nanoksa G-PLUS is evidently distinguished by its light weight, monomer-free nature, outstanding biocompatibility, (shock absorption) micro-mechanical elasticity, mechanical stability, remarkable strength characteristics, and long-term durability. Mimicking the natural teeth, the material offers high aesthetics, translucency and color stability. The manufacturer goal was to create a biomaterial of a high flexural strength comparable to that of *PEEK “Polyether ether ketone”* [18, 22].

Recently; Zirconia “ZrO_2_” is frequently used in dental restorative biomaterials due to its enhanced fracture resistance and improved tooth-like color. Incorporation of zirconia nanoparticles into the resin polymer matrix obviously increases the mechanical performance due to substantial ZrO_2_ filler strength and the excellent interfacial bonding [23]. Fortunately, it has been reported that addition of carbon fiber to the high-performance polymers reinforced it and enhanced their mechanical strength [24].

Evidently, one of the key points for construction of endocrowns is the suggested specific preparation design and adhesion guidelines. Endocrowns are essential in cases of extensive coronal destruction, where mechanical retention seems necessitous. When performing tooth preparation, preservation of the precious dental tissues as much as possible is considered critical; aiming to properly distribute the applied forces along the tooth itself and the coronal restoration instead of the adhesive bond interface [25]. The fundamental design of an endocrown relies on a cavity preparation where the intra-cameral or intra-radicular part will be integrated into the coronal tooth portion and extended in an apical direction. This protocol aims to provide a larger adhesive surface to improve the endocrown mono-block bonding interface [26]. Moreover, the design extension toward the pulp chamber should have at least a 2 mm depth. However, an extension of 3–4 mm may improve resistance of the endocrown, its catastrophic fracture tends unfortunately to be non-restorable [27]. Regardless the degree of tooth destruction, endocrown occlusal preparation should be minimum of 2 mm reduction; to obtain an adequate material thickness that achieve a uniform function of the restoration in a mono-block mode. Ideally, position of preparation margins should be perpendicular to axial forces, without any undercut for a safe distribution of applied stresses [28]. Inherently, the preparation has advantage of the mono-block design where; the stress distribution is extremely favorable for the biomaterial due to its thickness and volume [25, 29].

An alteration in the endocrown design is possible to improve aesthetics as well as the physio-mechanical characteristics of the delivered final restoration [12, 29]. Moreover, the composition of the restorative material has strongly influenced the preparation design, concerning the overall reduction in the occlusal height [18, 19]. Furthermore, when a ceramic mono-block material is used, the recommended tooth reduction in the axial direction should be minimum of 2 mm [30, 31]. However, from the occlusal plane an overall reduction of 1 to 1.5 mm was suggested in case of resin composite endocrowns due to the material stress-absorbing property and elastic modulus that might simulate those of human dentin [32, 33].

Successful serviceability and longevity of endocrown restorations are highly related to the biomaterial electronic configuration, adequate marginal adaptation, the RFT preparation design, and the applied adhesive bonding system [30, 31]. Therefore, the effect of two different endocrown biomaterials that are newly introduced in dental market: 3-D printed *Nano-zirconia*; Nano-carbon containing high performance polymer and *CAD/CAM* Hybrid Composite-Ceramic *“VITA ENAMIC”*, as well as the teeth-preparation designs (*Anatomic* and *Butt Joint*), on marginal adaptation and fracture resistance of posterior endocrown restorations were crucial issues for the clinical theater to be investigated in this in-vitro study.

Therefore, two null hypotheses were suggested: The 1st null hypothesis stated that the investigated Nanoksa G-PLUS and VITA ENAMIC endo-crown biomaterials had the same marginal gap distance (accordingly same marginal adaptation) and fracture resistance values. The 2nd one proclaimed that the applied preparation designs “Anatomic and Butt Joint” in each tested biomaterial had no effect on the marginal gap distance (accordingly marginal adaptation) and fracture resistance values.

## Materials and methods

### Ethical aspects

The design and procedures of the present study adhered to the World Medical Association (WMA) Declaration of Helsinki, [https://www.wma.net/policies-post/wma-declaration-of-helsinki/]. The 40 periodontically affected sound teeth used in the study were indicated for essential extractions (as foci of infection in patients’ body). The patients were informed about the detailed use of only the hard tissues of extracted teeth for that in vitro research studies and no one had reported any objection about it. A written consent was routinely filled and signed by each patient. In addition, the study details were accomplished following the research guidelines that were adopted by the research ethics committee, Faculty of Dentistry, Menoufia University, Egypt and the ethical approval number was ADMNF-01225.

### Materials

In the current study, the biomaterials used for fabrication of endocrowns, the applied adhesive systems and adhesive resin cements were explained in (Table 1). VITA ENAMIC is a novel commonly used hybrid dental ceramic. Nanoksa G-PLUS is a strictly standardized commercial product, an experimental nano-reinforced polymer composite formulation. VITA ENAMIC is a fine-structure ceramic network (86wt.%, 75 vol%) strengthened with an acrylate polymer network (14 wt%, 25 vol%) that completely fills the ceramic’s pores. Therefore, the two networks are fully integrated within each another. Moreover, the VITA ENAMIC polymer material network consists of UDMA and TEGDMA surface-modified PMMA that is MMA free (Table 1).

**Table 1:** Endocrown ceramic biomaterials and adhesives

Commercial Product	Classification	Manufacturer	Composition
Nanoksa G-PLUS	Polymers and Nano-Zirconia Fiber	INOX (Nanoksa G -PLUS)	High performance polymers containing Nano Zirconia a G -Plus and carbon for production of permanent restorations.
Z-Prime PLUS Zirconia	Zirconia Primer	Schaumburg, Illinois, USA	Two active monomers: MDP; a phosphate monomer, and BPDM; a carboxylate monomer.
VITA ENAMIC	Hybrid Ceramic	VITA Zahnfabrik, Germany	*Inorganic phase*: A fine feldspar ceramic structure (86wt.%, 75 vol%) strengthened with a porous structure infiltrated by a polymer. *Organic phase*: A methacrylate polymer (14 wt%, 25 vol%) networks consisting of UDMA and TEGDMA surface-modified PMMA that MMA free that reinforces and strengthens the ceramic base. *Whole structure*: The VITA ENAMIC biomaterial is a polymer-infiltrated ceramic network “PICN”.
VITA ENAMIC Ceramic Etch	Ceramic Etch	VITA Zahnfabrik, H. Rauter GmbH Bad Sackingen, Germany	5% Hydrofluoric Acid Gel
Silane Ultra Dent	Silane Coupling Agent	Ultradent Product, INC, USA	Organo-silane (MPS) 5–15%, Isopropyl alcohol 92%, Acetic acid < 1%
Breeze™	Self-Etch Adhesive Resin Cement	PentronN, UK	N97Breeze, Self-Adhesive Cement Intro Kit, 3–4 ml syringes A2; 1–4 ml syringe Translucent; 1–4 ml syringe Opacious White; 1-3 ml Bottle Silane; 40- Auto-Mix Tips; 10-Intra-Oral Auto-Mix Tips.

## Methods

### Study design

#### Study setting

The current in-vitro study was conducted on very loose human permanent molars in private clinics extracted because of periodontal diseases, and the used in vitro tests were carried out in material laboratories.

#### Sample size

The study sample size was calculated using a computer program G-power version 3.1.9. Evidently; the significance level was 0.05, the obtained power sample size was more than 80% and the confidence interval was 95%.

#### Formula of sample size [33]

$$Sample\,Size=\left({\sigma}_{1}^{2}+{\sigma}_{2}^{2}\right){\left({Z}_{\alpha/2}+{Z}_{\beta}\right)}^{2}/{\left(\varDelta-{\varDelta}_{0}\right)}^{2}$$

Where:

𝜎_1_^2^, 𝜎_2_^2^ = variance of the 2 groups.

𝑍_𝛼/2_  = Type I error.

𝑍_𝛽_ = statistical power.

Δ = Expected difference between means.

Δ_0_ = Difference under the null hypothesis.

#### Specimens selection

Human intact 40 mandibular permanent molars that were essentially extracted (as foci of infection in patients’ body) because of excessive mobility, and periodontal affections were collected and used in the in vitro study. All study teeth had sound completed root formation, without any fracture line or resorptive defect. All molars were soaked for 10 min in 5% Sodium Hypochlorite, then, thoroughly cleaned from calculus and all soft tissue debris using an ultrasonic scaler (Woodpecker, China). All specimens were individually stored under identical conditions; in a saline solution at 5 °C for up to six months to avoid its dehydration. Afterword; throughout the study, the saline solution was weekly refreshed according to the International Organization instructions.

#### Endodontic teeth treatment

The coronal access cavities were performed using round burs (No.271) at water-cooled high-speed while following morphology of the pulp chamber in each molar. The root canal system was cleaned and shaped, and obturated using Gutta-percha coated with a resin sealer (ADSEAL, Meta-Biomed, Korea LOT ADS2104141).

#### Specimens preparation

A cylindrical plastic mold with an internal diameter of 19 mm and 2 mm was filled with auto-polymerizing acrylic resin (Acrostone, Cairo, Egypt) and before its set the roots of the study molar was vertically inserted and centralized inside the resin at 2 mm just below the cement enamel junction (CEJ) using a dental surveyor (Marathon-103, Saeyang Company). Consequently, the teeth blocks were prepared for each study molar [34].

#### Samples grouping

The study mandibular molar teeth were endodontically treated. According to the fabricated endo-crown biomaterial, study samples were classified into two main groups; Nanoksa G-PLUS and VITA ENAMIC, (*n* = 20 each). Afterward; each group was divided according to the preparation design (anatomical and butt joint designs) into two subgroups (*n* = 10 each).

- Group 1 (G1): Nanoksa G - PLUS Endocrown; (*n* = 20)
  - ➢Subgroup A; Nanoksa G -PLUS endocrown with an Anatomical design preparation; (G1A) (n = 10).
  - ➢Subgroup B; Nanoksa G - PLUS endocrown with Butt joint design preparation; (G1B) (n = 10).
- Group 2 (G2): VITA ENAMIC Endocrown *(Hybrid Ceramic)*; (*n* = 20)
  - ➢Subgroup A; VITA ENAMIC endocrown with an Anatomical design preparation; (G2A) (n = 10).
  - ➢Subgroup B; VITA ENAMIC endocrown with Butt joint design preparation; (G2B) (n = 10).

#### Standardization of tooth preparations for ceramic endocrowns

All specimens were prepared by a single operator and a computerized numerical control (CNC; C.N.C Premium 4820, imesicore, Eiterfeld, Germany) milling machine was used for performing the selected anatomic and butt joint preparation designs; in order to standardize uniform preparation dimensions for all molars in each subgroup. The milling machine diameter was 3 mm, maximum spindle speed was 28,000 rpm, spindle taper type as ISO SK20, positioning accuracy was ± 0.01, cooling method was: Oil Mist for (Wet) / Air and Suction for (Dry) and diamond-coated (DLC/Multi-layer).

**Fig. 1:**
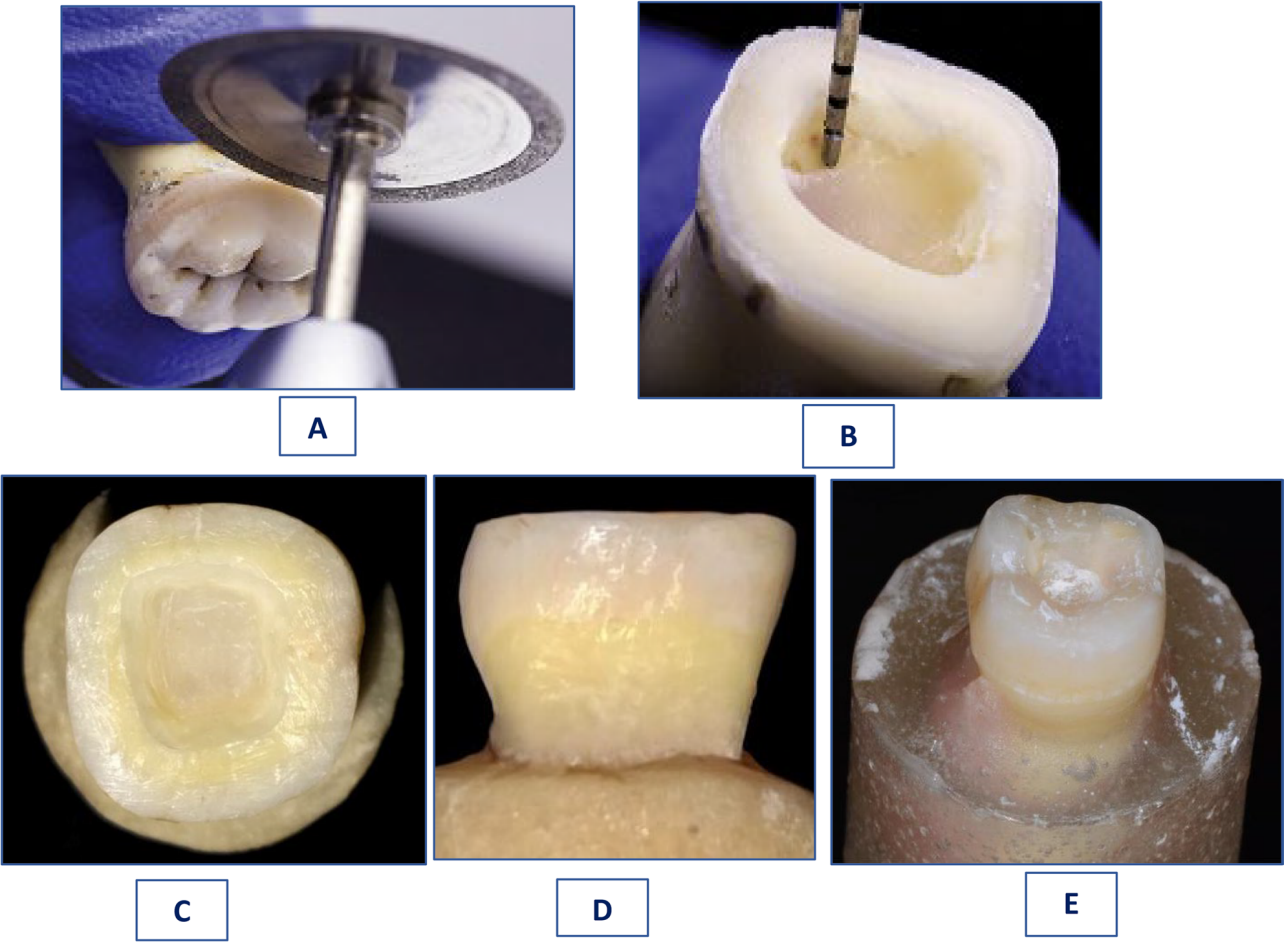
**(A)** Drawing of 3 Lines on Tooth Surfaces to Precisely Determine the Dentin Bridge Thickness, **(B)** Graduated Periodontal Probe to Standardize the Cavity Depth, **(C)** Occlusal View of a Butt Joint Preparation Design, **(D)** Profile View of a Butt Joint Preparation Design, and **(E)** Occlusal View of an Anatomic Preparation Design

Occlusal surfaces of all specimens were reduced by 2 mm in axial direction using diamond wheel bur (3054-024-21.0, Microdont, USA) to create a 360° butt-joint surface. Complete flat surface was ensured by orienting the bur along the tooth long axis and parallel to the occlusal plane. All sharp internal line angles were smoothed and rounded with fine-grit (30–40 μm) tapered diamond bur (4137 F-856-025-L1 8.0-L2 21.0. Microdont, USA) [35]. Furthermore; a graduated standardized periodontal probe helped to properly adjust 3 mm depth of the internal cavity. Concerning the anatomic design of A subgroups in both group 1 and 2 (G1A & G2A), the occlusal reduction was done following anatomy of the occlusal tooth surface (Fig. 1: A, E) [35]. Regarding the butt joint design in subgroups B for both group 1 and 2 (G1B & G2B), a diamond coarse wheel directed parallel to the occlusal plane was used to achieve a horizontal smooth flat margin after performing the preparation (Fig. 1: C, D).

Apropos of preparing the internal cavity, it was initiated by removal of all present undercuts, roundation of all internal line as well as point angles and elimination of probable recesses found in the pulp chamber while still maintaining its original morphology. Moreover; internal divergent (8–10° taper degree) walls were obtained using a tapered diamond cutting bur grasped perpendicular to pulpal floor. Furthermore; a graduated standardized periodontal probe helped to properly adjust 3 mm depth of the internal cavity (Fig. 5: B). Finally; standardization and adjustment of all cavity margins at a uniform thickness (~ 3 ± 0.5 mm) was attained by a digital caliper (New Med, Pakistan) [36].

#### Designing endocrown restorations

**Fig. 2:**
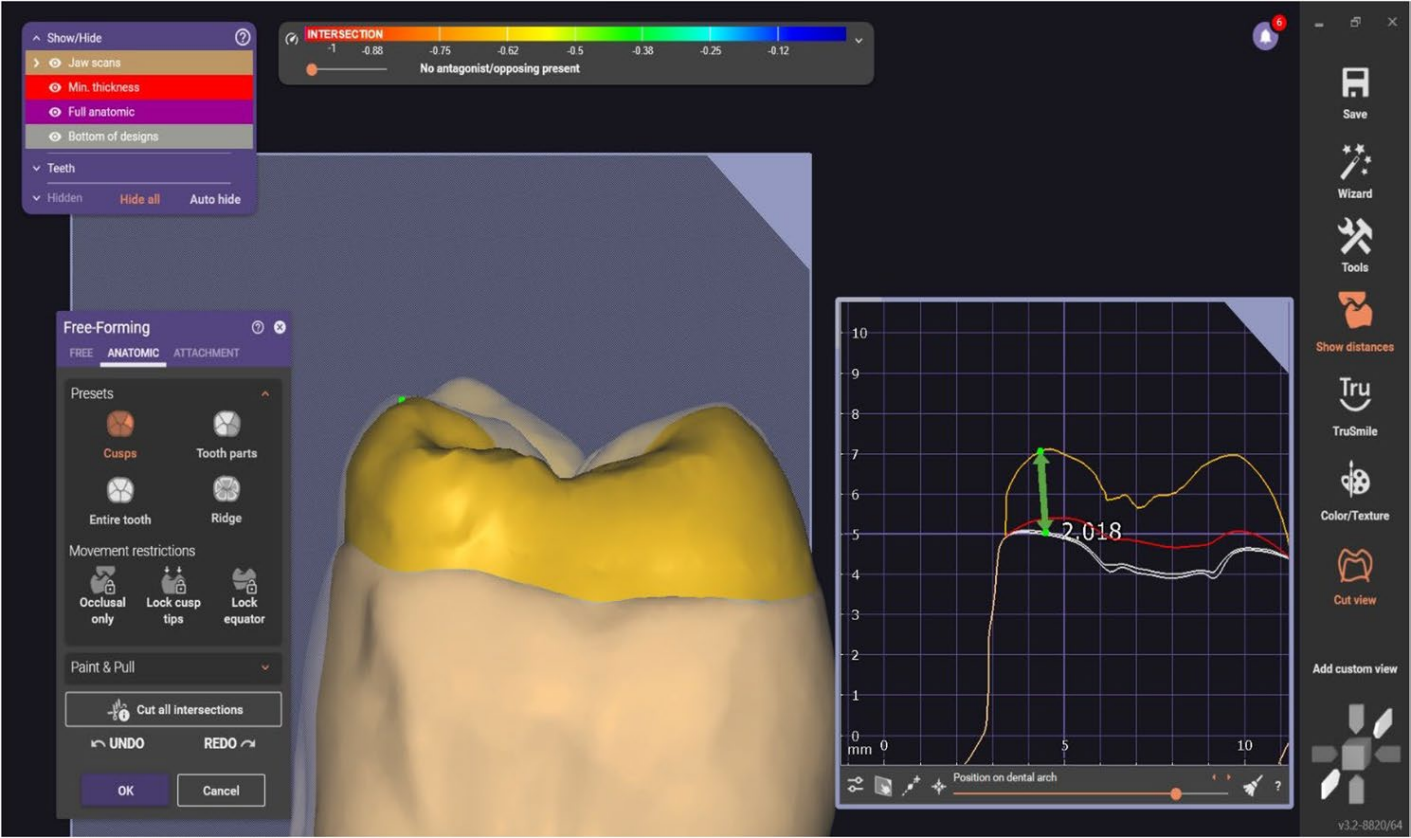
An endocrown exocad for an anatomic preparation design

**Fig. 3:**
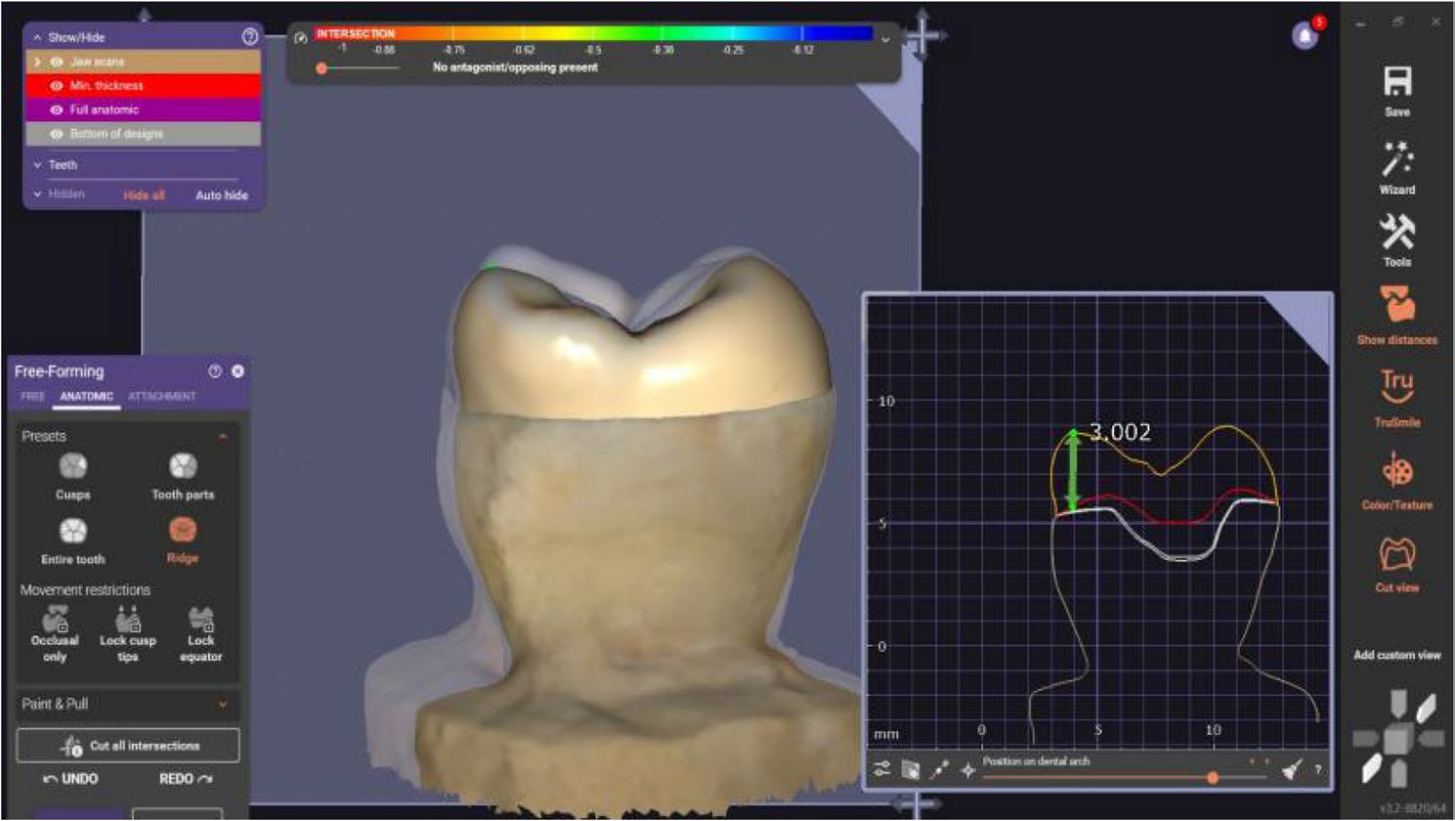
An endocrown exocad for a butt joint preparation design

After selecting the group design and matching the tooth number using the software exocad program (Exocad GmbH, Darmstadt, Germany), the endocrowns were individually tailor-made for each molar using the standardized measurements. The software system was implemented to obtain a three-dimensional image of the prepared molar as the study tooth was sprayed by Telescan light reflection powder (Vita Zahnfabrik, Germany) to produce an optical impression of the tooth sample. Furthermore, scanning using identical blue with the automatic marginal finder accurately determined the preparation margins [37, 38]. Therefore, the STL file was created for milling of the endocrown material using a CAD/CAM machine (Fig. 2, and 3).

#### 3D printing of nanoksa G-PLUS endocrown

The 3D Printing of Nanoksa G-PLUS preparation procedures concerned gradual addition of nanoparticle’s proportion into the solution of printable resin whilst; continuously blending on a magnetic stirring machine for 24 h. Afterward, the resin solution underwent sonication in a distilled water bath for 30 min. Aiming to 3D print by the same setup with a DLP printer (Phrozen Sonic Mini 4 K, Hsinchu 30091, Taiwan), a light source (LED; of 385 nm wavelength) was used. Adhering to manufacturer’s guidelines, the produced 3D printed endo-crown specimens were soaked for 5 min in 90% isopropyl alcohol. Then, the specimens were carefully separated from the building platform by aid of a scraper. Afterword; a second rinse was performed using fresh isopropanol to remove any trace of uncured monomer from the material surface. Finally; the obtained specimens were dried with compressed air. In addition, the polymerization process was completed using a post-curing treatment for 10 min, aided by a light curing device (Broad wavelength spanning spectrum of 400–550 nm Solidilite V, Shofu Dental GmbH, Ratingen, Germany).

#### VITA ENAMIC endocrown fabrication using CAD/CAM technology

Before scanning, each prepared molar was well sprayed with a special optical reflecting substance (Telescan light-reflecting powder, VITA Zahnfabrik, Germany); in order to produce a 3D digital picture. Afterword; these teeth were again scanned with a Smart scanner (DOF Freedom UHD lab scanner) and the obtained captured scans were saved to the CAD/CAM software. All study specimens were sequentially fixed on the scanner tray for scanning and final optical impressions were obtained.

After fixation into the computer controlled milling unit (CORITEC350i loaderpro), CAD/CAM software had designed the endocrowns and under a full automation, the milling machine manufactured it. Moreover, VITA ENAMIC designed endocrowns were performed with comparable occlusal anatomy and height.

#### Treating the intaglio surfaces of endocrowns

- Group1 (G1): Nanoksa G - PLUS Endocrown; (*n* = 20)

Two preparation designs:

- ➢Subgroup A; Nanoksa G -PLUS endocrown with an Anatomical design preparation; (G1A) (*n* = 10)
- ➢Subgroup B; Nanoksa G - PLUS endocrown with Butt joint design preparation; (G1B) (*n* = 10)

**Fig. 4:**
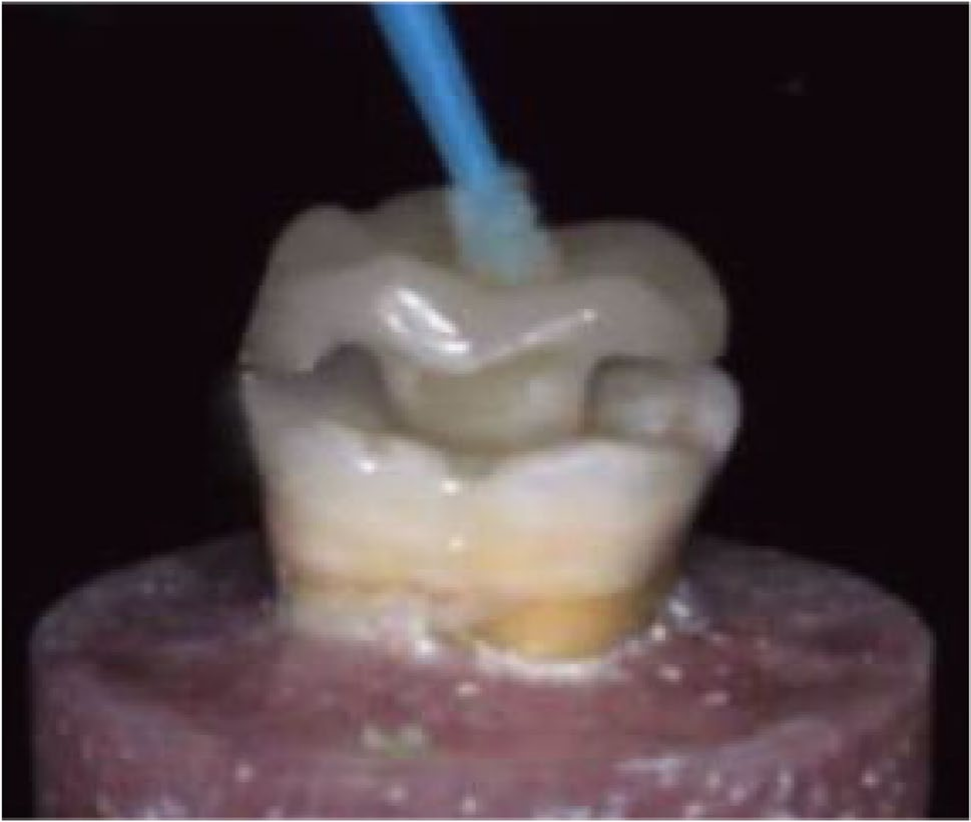
Try-in of a Nanoksa G-PLUS Endocrown Restoration with an Anatomic Preparation Design

Try-in of Nanoksa endocrown was done to ensure the accurate fit of restoration. Accordingly, intaglio surfaces of each specimen restoration were sandblasted from a 10 mm distance using 30–50 μm aluminum oxide ″**Al**_**2**_**O**_**3**_**″** particles for 10 s with 2.5 bar pressure (Fig. 4). Consequently, each restoration was properly debrided in an ultrasonic cleaner bath or in an alcohol rinse. Then, the restoration was completely air dried to receive at the inner surface an applied Silane Coupling Agent (Silane Ultra Dent) that was essential to promote the chemical bonding [36].

- Group2 (G2): VITA ENAMIC Endocrown Hybrid Ceramic; (*n* = 20)
  - ➢Subgroup A; VITA ENAMIC endocrown with an Anatomical design preparation; (G2A) (*n* = 10)
  - ➢Subgroup B; VITA ENAMIC endocrown with Butt joint design preparation; (G2B) (*n* = 10)

**Fig. 5:**
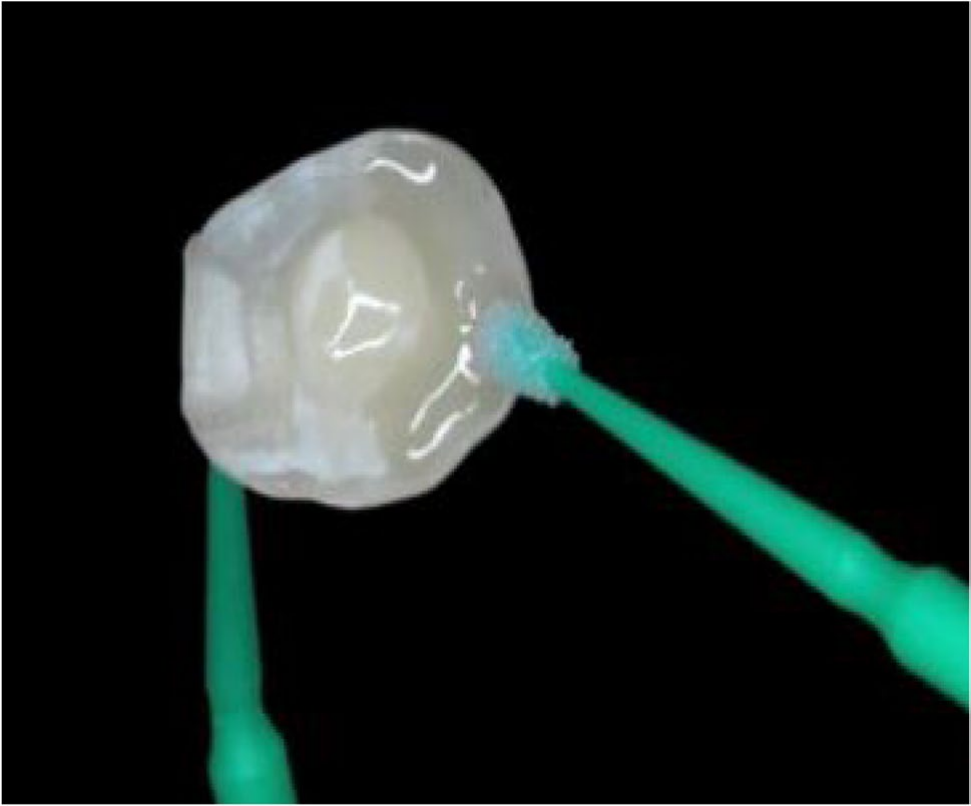
Etching Inner Surface of VITA ENAMIC Endocrown Restoration with Hydrofluoric Acid

Following the manufacturer instructions, inner surface of each VITA ENAMIC endocrown was etched with 9.5% hydrofluoric acid for 20 s, then, properly rinsed for another 20 s and well dried (Fig. 5). Afterword, a recommended silane bonding agent (e.g.; VITA ADIVA C-PRIME) was applied to the surface treated VITA ENAMIC endocrown [36, 39].

#### Cementation of endocrown restorations

The manufacturer instructions were strictly implemented with each endocrown material as well as each adhesive resin cement used in the study. Following the manufacturer guidelines, apply an appropriate amount of adhesive resin cement (dual- or self-cure resin cement with phosphate monomers), (SoloCem, Coltène/Whaledent AG, Switzerland). The resin cement was applied into the fitting surface of the endocrown with a gentle seating pressure on the molar tooth, till the complete restoration seating was achieved. A cementing device with a load of 3- kilogram was applied on the specimens for five minutes only after cement application to standardize an equally distributed homogenous thickness of cement layer under equivalent pressure, and then any excess cement was removed with a brush. Furthermore; to achieve a proper setting procedure, a thin layer of glycerin gel was placed on the restoration margins to hinder the oxygen inhibitory effect during cement polymerization. Finally; photo-curing of the surface for 20 s was done using a blue phase light curing device (Ivoclar Vivadent AG, Schaan, Liechtenstein).

#### Thermocycling and chewing simulation

Aiming to simulate the intraoral conditions, all the obtained molar blocks with the cemented endocrowns were individually subjected to a chewing simulator with thermal cycles CSTC device (CS-4, SD Mechatronik, Germany). Main thermocycling chamber of used chewing simulator consisted of four testing chambers. Essentially, all study specimens were subjected to 12,000 chewing cycles, 1 min/cycle; (corresponding to approximately 1.2 year of the in vivo function) applying a 50 N compressive load at a frequency of 1.6 Hz and temperature range 10–60 °C. The chewing simulator had two movable parts; one with a vertical axis and the other with a horizontal one. The specimens were mounted on a mobile table that oscillated back and forth; while a customized antagonist (4 mm diameter) was connected to the vertical bar that was moving up and down in association with a 5 Kg load in order to simulate the intra-oral masticatory stresses [40, 41].

#### Assessment of marginal gap distance

**Fig. 6:**
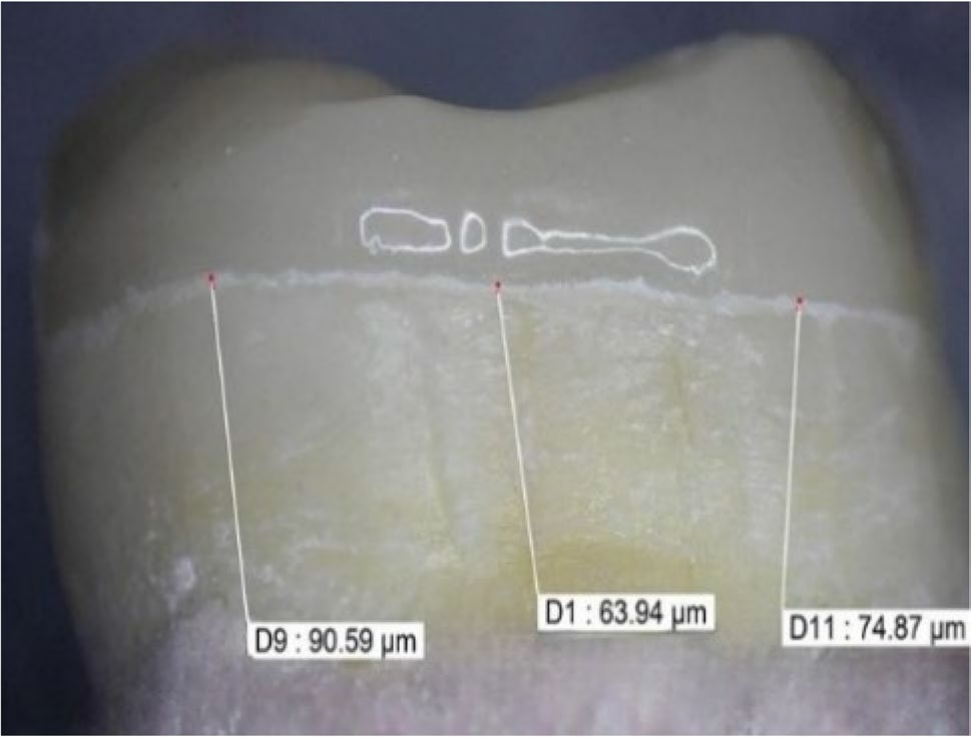
Margin Adaptation Assessment of an Anatomic Endocrown by a Stereomicroscope

Each group specimen was photographed using a USB digital stereomicroscope containing a built-in camera (U500x Digital Microscope, Guangdong, China) to produce a high image acquisition. Moreover, for each molar and on every tooth surface, a small round bur was used to create and fix three equidistant marks at 1 mm from the endocrown margin to standardize the location of the measurements’ points for all study specimens. Furthermore; for each specimen shots of the sample’s margins were taken. Consequently; the morphometric measurements were obtained for each shot that was 2–3 equidistant landmarks along each specimen surface circumference (Fig. 6).

In addition, the marginal gap width was measured and evaluated by aid of a digital image analyzing system (Image J 1.43U, National Institute of Health, USA); where all measured parameters, sizes, frames, and limits were expressed in pixels within the Image J software. Therefore, the system was calibrated to convert the reported pixels into real absolute world unit of length; where one in “inch"= 2.54 cm = 96 px “pixel” and one px ~ 0.0265 mm [2.54/96] [42]. Evidently; software calibration was implemented by comparing an object (considered as a ruler in the study work) of known dimensions with a scale that was generated by Image J software. Then, the obtained system data were collected, tabulated for subjection to statistical analysis [42]. The high marginal gap values indicate a poor marginal adaptation while the low values denote a good marginal adaptation at tooth-restoration interface.

#### Fracture resistance test

**Fig. 7:**
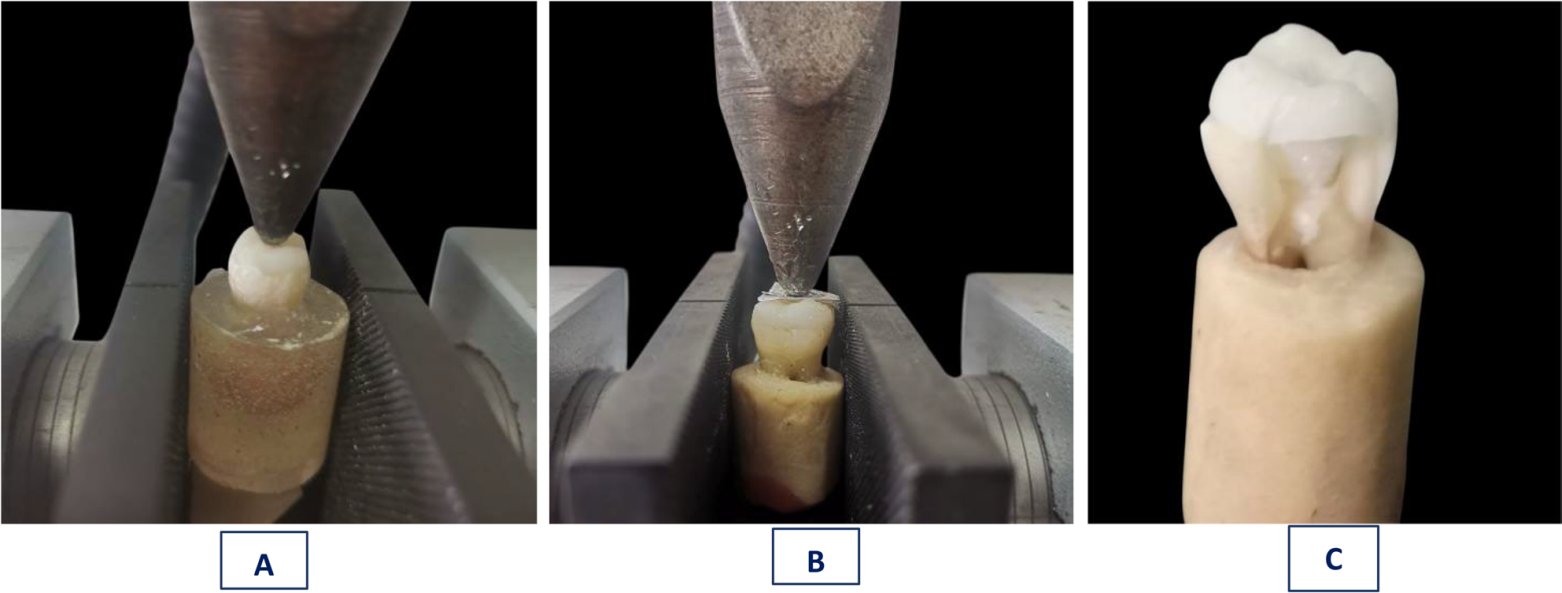
Fracture Resistance Test Using a Universal Testing Machine **(A)** Failure of an Anatomically Designed Endocrown, **(B)** Failure of a Butt-Joint Designed Endocrown, and **(C)** Failure of Specimen (Tooth with Endocrown) after Fracture Test

Each specimen was mounted within the lower compartment of a universal testing machine (Model 3345; Instron Industrial Products, Norwood, MA, USA) and it was subjected to an increasing static compressive load till its failure. A stainless-steel ball (6 mm diameter, and 5Kn load cell) loading piston was used to vertically apply the force to the occlusal surface till the complete fracture (Fig. 7). Then, the fracture load readings were recorded in Newton using a specific computer software (Instron Bluehill Lite Software) [42].

### Statistical analysis

Data analysis was performed using the Statistical Package for Social Sciences (IBM SPSS Statistics version 26). Numerical variables were expressed as the mean “M” and standard deviations “± SD”. The P value < 0.05 was considered a statistically significant difference. Data was tested for normality using the Shapiro-Wilk normality test. A two-way ANOVA followed by Tukey’s post-hoc tests were utilized for determining the statistical difference among the test groups to test the effects of the factor “material”, the factor “design”, and the interaction between both factors and to detect if there was a statistically significant difference in fracture resistance and marginal gap values of the study subgroups [43].

## Results

The obtained data were fed to a computer for statistical analysis using IBM SPSS software package version 20.0. (Armonk, NY: IBM Corp, released in 2011), where qualitative data were described using numbers and percent. The Shapiro-Wilk test was used to verify the approximate normality of data distribution. Quantitative data were described using mean and standard deviation. Significance of the obtained results was judged at the 5% level. A two-way Analysis of Variance ″ANOVA″ (Material ×Design) followed by Tukey’s post-hoc tests were applied to compare each two independent study subgroups; where all obtained study results were expressed by statistical means “M”, standard deviations “± SD”.

**Fig. 8:**
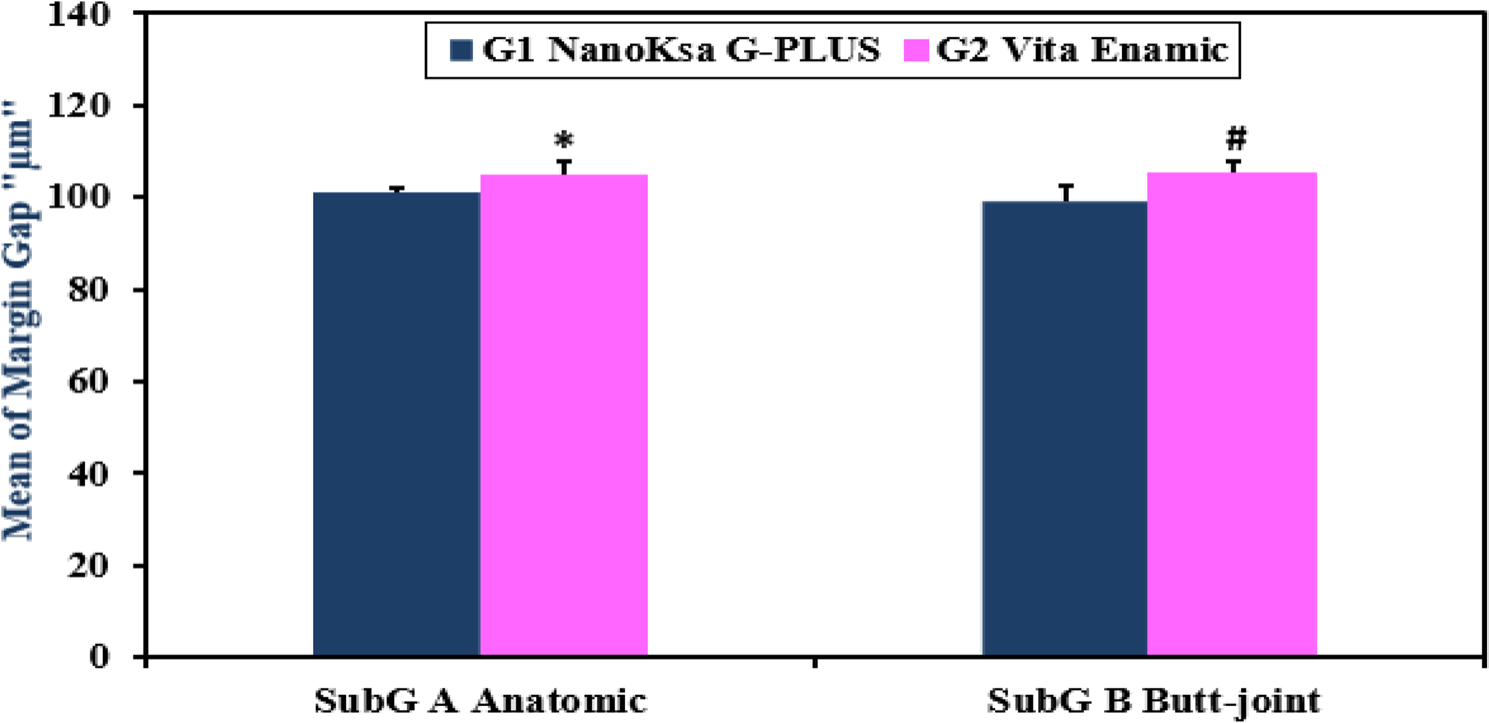
Comparing Margin Gap Mean Values “µm” of the Different Study Endocrown Groups

Concerning assessment of endocrown marginal gap distance, a two-way ANOVA test was conducted to investigate the effects of endocrown restorative materials (NanoKsa G-PLUS vs. VITA ENAMIC) and tooth preparation design (anatomic vs. butt joint) on the margin gap values (µm). Where high mean values elucidated a poor marginal adaptation and low mean values indicated a successful good marginal adaptation, the G2B " VITA ENAMIC endocrown of a butt-joint design preparation” showed highest value (105.3 ± 2.41 μm), followed by G2A " VITA ENAMIC endocrown of an anatomical design preparation” (104.87 ± 3.07 μm), then, G1A “Nanoksa G -PLUS endocrown for an anatomic design preparation” (101.0 ± 1.14 μm) and the least was G1B “Nanoksa G -PLUS endocrown for a butt joint design preparation” (99.25 ± 3.31 μm) (Table 2; Fig. 8).

Consequently, the implemented two-way ANOVA test revealed a significant main effect of endocrown biomaterial (F _(1, 36)_ = 35.80, *P* < 0.0001), and it did not detect any significant main effect of “ETT” preparation design (F _(1, 36)_ = 0.6339, *P* = 0.4312) nor a significant interaction between both study factors (F _(1, 36)_ = 1.729, *P* = 0.1969). Therefore, the investigated Nanoksa G -PLUS endocrown material reported relatively better marginal adaptation mean values (least marginal gap values) compared to those of VITA ENAMIC endocrown for all applied preparation designs “i.e. anatomic and butt-joint” compared to its respective subgroup, (Table 2; Fig. 8).

Therefore, 1st null hypothesis that suggested no difference in marginal gap distance mean values and marginal adaptation between the investigated Nanoksa G-PLUS and VITA ENAMIC endocrown biomaterials was evidently rejected. In addition, the 2nd one that proclaimed that the applied preparation designs “Anatomic and Butt Joint” in each tested biomaterial had no effect on marginal gap distance mean values and marginal adaptation was accepted for VITA ENAMIC and Nanoksa G-PLUS endocrowns.

**Table 2:** Comparing margin gap mean values of the different study groups

	Group 1 (NanoKsa G-PLUS) “µm” Mean ± SD	Group 2 (VITA ENAMIC) “µm” Mean ± SD	Two-way ANOVA		
			Endocrown Material	Preparation Design	Interaction
Sub A (Anatomic)	101.0 ± 1.14	104.87 ± 3.07^*^	F (1,36) = 35.80 *P* < 0.0001	F (1,36) = 0.6339 *P* = 0.4312	F (1,36) = 1.729 *P* = 0.1969
Sub B (Butt Joint)	99.25 ± 3.31	105.3 ± 2.41^#^			

Data was expressed as mean ± SD (n = 10). ^*^P value significant versus the G1 NanoKsa G-PLUS/Sub A (Anatomic) group, ^#^*P* value significant versus the G1 NanoKsa G-PLUS/Sub B (Butt joint) group. Treatments were compared by two-way ANOVA followed by Tukey's post-hoc test

**Fig. 9:**
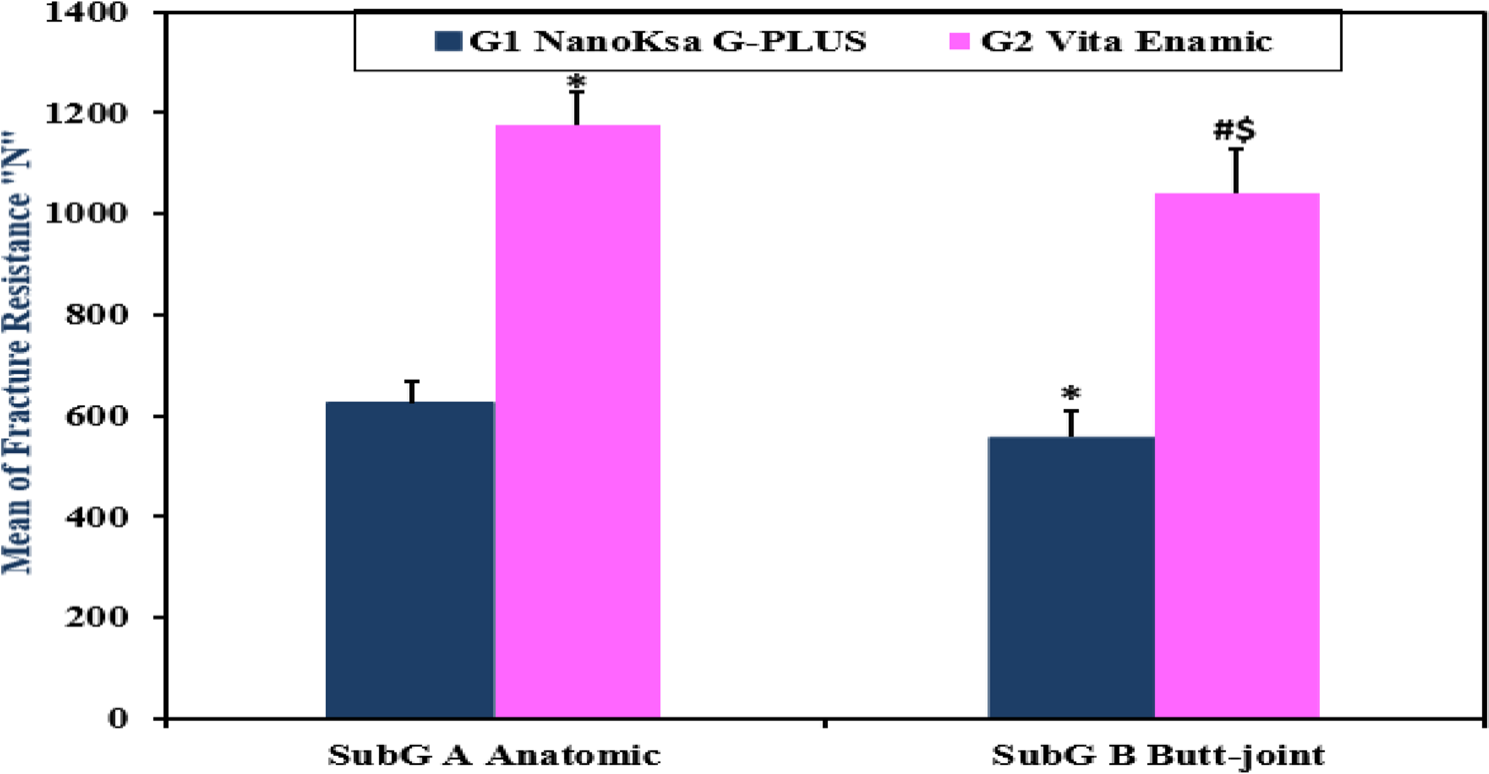
Comparing Fracture Resistance Mean Values (in Newton “N”) of the Different Study Endocrown Groups

Regarding statistical analysis of fracture resistance, the conducted two-way ANOVA test was performed to evaluate influence of endocrown material (NanoKsa G-PLUS vs. VITA ENAMIC) and the molar preparation design (Anatomic vs. Butt Joint) on fracture resistance (measured in Newtons, “N”) of the research specimens. Evidently, G2 “VITA ENAMIC endocrown reported highest fracture strength mean values in the two applied preparation designs “G2A, G2B” that were 1176.4 ± 66.58 N, 1040.4 ± 86.91 N; respectively. However, the least fracture resistance mean values were shown for G1 “Nanoksa G -PLUS” in the two molar preparation designs “G1A, G1B” that were 625.2 ± 44.05 N, and 557.6 ± 52.42 N; respectively (Table 3, Fig. 9).

Accordingly, two-way ANOVA revealed significant main effects of both endocrown biomaterial (F _(1, 36)_ = 641.2, *P* < 0.0001) and the tooth preparation design (F _(1, 36)_ = 24.86, P < 0.0001) and it did not show any statistically significance for the interaction between the study factors (F _(1, 36)_ = 2.806, P = 0.1026). Notably, VITA ENAMIC subgroups “i.e. Anatomic and Butt joint” showed a higher fracture resistance mean values compared to their respective Nanoksa G -PLUS subgroups, and in the same direction, subgroups A “Anatomic” showed a significant increase in fracture resistance mean values compared to their respective subgroups B “Butt joint” (Table 3, Fig. 9).

Therefore, the 1st null hypothesis that declared that the investigated Nanoksa G-PLUS and VITA ENAMIC endocrown biomaterials had no significant difference in fracture resistance values was declined. The 2nd one that proclaimed that the applied preparation designs “Anatomic and Butt Joint” in each tested biomaterial had no effect on fracture resistance values was also rejected for both Nanoksa G-PLUS and VITA ENAMIC endocrown groups.

**Table 3:** Comparing Fracture Resistance Mean Values (in Newton “N”) of the Different Study Groups

	Group 1 (NanoKsa G-PLUS) “µm” Mean ± SD	Group 2 (VITA ENAMIC) “µm” Mean ± SD	Two-way ANOVA		
			Endocrown Material	Preparation Design	Interaction
Sub A (Anatomic)	625.2 ± 44.05	1176.4 ± 66.58^*^	F (1,36) = 641.2 *P* < 0.0001	F (1,36) = 24.86 *P* < 0.0001	F (1,36) = 2.806 *P* = 0.1026
Sub B (Butt Joint)	557.6 ± 52.42^*^	1040.4 ± 86.91^$#^			

Data was expressed as mean ± SD (*n* = 10). ^*^P value significant versus the G1 NanoKsa G-PLUS/Sub A (Anatomic) group, ^**#**^*P* value significant versus the G1 NanoKsa G-PLUS/Sub B (Butt joint) group, ^**$**^*P* value significant versus the G2 VITA ENAMIC/Sub A (Anatomic) group. Treatments were compared by two-way ANOVA followed by Tukey’s post-hoc test

## Discussion

Endocrowns evidently present a unique concept for a minimally invasive *ETT* restoration that is not only used to restore but also to protect *ETT*, with no need to sacrifice the intra-radicular structure for retention of the post and core system. Endocrown treatment modality relies mainly on the macro-mechanical retentive features through its extension into pulp chamber cavity (large surface area) as well as, micro-mechanical retention and chemical bonding with the new adhesive systems. Consequently, maximum preservation of the precious remaining sound tooth structure seems nowadays achieved; due to the obtained advantages of recently developed smart technologies in bio-ceramic materials, endocrown construction techniques, and the innovated adhesive resin bonding systems [43, 44]. Indeed, biomechanical properties of restorative dental prosthetic materials play a critical role in serviceability, success, and durability of post-endodontic coronal restorations and consequently the clinical persistence of *ETT*. Endocrowns appear as novel high-performance dental restorations, they are described as adhesive monolithic ceramic restorations, retaining micromechanically to the pulp chamber border assets, and anchoring in the pulp chamber [45, 46].

In addition, various endocrown materials were introduced as an alternative conservative treatment modality for *ETT*. Relatively, the endocrown technique is supra-gingival facilitating clinical inspection, and plaque control, less chairside time, easy, and cost effective. Recently, endocrown smart new technologies allow strengthening of *ETT* with a minimal tooth reduction and preservation of remaining coronal and radicular dental structures. Concerning clinical procedures, endocrown restorations for many levels are proven to be simpler, easier and faster than traditional post, core and crown procedures [47, 48]. Therefore, various novel biomaterials and high technologies were developed for endocrowns, including *3D* printed, *CAD/CAM* “*Computer Aided Design/Computer Aided Manufacturing”* systems and software using reinforced polymeric and ceramic biomaterials; offering several clinical advantages [48, 49].

Recently, resin infiltrated bio-ceramics “ceramics incorporated within a resin matrix” were newly introduced in the dental market, aiming to compensate the resilience of lost coronal dentin due to their comparable modulus of elasticity and resistance to fracture to those of natural dentin. Fortunately, these developed biomaterials decreased catastrophic failure rate by proper stress distribution, and improved fracture resistance.

Consequently, in the current study two types of endocrown materials; a resin infiltrated high-performance polymer; *“Nanoksa G -PLUS”* and a hybrid ceramic; *“VITA ENAMIC”*, were selected to evaluate their impact on stress distribution, resistance to crack propagation and marginal adaptation to *ETT* coronal restorations. Nanoksa G-PLUS as a high-performance polymer, and VITA ENAMIC as a hybrid ceramic seem admirable smart recently innovated composites that combine the pleasant aesthetic appearance and high strength of ceramic dental biomaterials with resiliency of an acrylate polymer matrix that infiltrate these present material’s pores.

Biocompatible Nanoksa G-PLUS composition is a blend of high-performance polymeric materials mainly *“PMMA”* " polymethyl methacrylates” incorporating Nano-zirconia and Nano-carbon fillers for fabrication of permanent *Class II* teeth restorations and prosthetic restorations of dental implants. Functionally, that material has a high cutting performance, an excellent abrasion resistance and a long-term durability. In addition, developed Nanoksa G-PLUS is designed to provide translucency, and high aesthetics mimicking the natural teeth color parameters *“hue*,* value and chroma”* [50].

In recent years, VITA ENAMIC has gained popularity as a hybrid ceramic veneering material for single-tooth restoration. Compared to ceramics, CAD/CAM composites reported lower stiffness *“moduli of elasticity”* and micro hardness values; making them relatively easier for use with the milling machines. Therefore, these findings were clinically beneficial for the opposing dental hard and soft tissues that would be probably subjected to lesser surface wear and damage [51].

All freshly extracted human permanent molars were stored under identical conditions to minimize the comparative bias between subgroups. Molars were used in that in vitro study; aiming to simulate the clinical theater, regarding the bonding of enamel and dentin through an adhesive resin system to the newly developed hybrid bio-ceramics. Accordingly, the specimens were prepared to evaluate the fracture resistance properties of the hard-dental tissues and investigate the dissipation of stresses on the whole tooth complex resembling an assumed real clinical situation. However, the human teeth might inevitably provoke some degree of variation in obtained study results because of the complex standardization of teeth preparations [52, 53].

The 1st study null hypothesis was comparatively rejected concerning the material factor as the obtained results showed a highly significant difference in marginal adaptation (evaluated from the marginal gap distance mean values) [F (1,36) = 35.80, *P* < 0.0001] and the fracture resistance mean values [F (1,36) = 641.2, *P* < 0.0001] between G1 “Nanoksa G-PLUS” and G2 “VITA ENAMIC” endocrown restorative materials; (Tables 2 and 3; Figs. 8 and 9). VITA ENAMIC reported higher fracture resistance than Nanoksa G-PLUS. Fortunately, “VITA ENAMIC” endocrown specimens prepared with anatomic design showed a considerable improvement in fracture resistance compared to those assigned with butt joint design. As a recent ceramic material developed by new technology, VITA ENAMIC is inherently characterized by high degree of crystallinity that markedly increases all the mechanical properties including the Young’s modulus, hardness, toughness, and fracture resistance. Those findings were in strong agreement with Asmaa Abd El Rhman, Mohamed El Layeh and Mohamed Hamed Ghazy [47] who assessed the retention and fracture resistance of various *CAD/CAM* endocrowns materials cemented for maxillary premolars’ restoration. Evidently, these hybrid ceramic biomaterials showed fracture resistance mean values comparable to those the natural teeth and endocrown materials; which was probably attributed to the manufacturers’ technology of the developed polymer network microstructure incorporated within the biomaterial ceramic matrix, aiming to adjust its elastic modulus, increase the fracture toughness and its resistance to crack propagations [50, 54].

Endocrown preparation design also showed a statistically significant main effect on fracture resistance [F (1,36) = 24.86, *P* < 0.0001], therefore; specimens prepared following the anatomic design exhibited higher fracture resistance mean values than those applying the butt joint design within both studied material groups (Table 3; Fig. 9). That finding related to “Nanoksa G-Plus” was convenient with Rami et al [44], might be due to the inherent resiliency of the new developed “Nanoksa G-Plus” acrylate polymer matrix containing Nano-zirconia fibers “a high percentage of 86%” and Nano-carbon that infiltrate the present material porosity. The study limitations concerning type, thickness and adhesive bond strength of applied resin cement with “Nanoksa G-PLUS” showed be acknowledged.

Meanwhile, a statistically significant difference in fracture resistance mean values was detected between the two preparation “anatomic and butt joint” subgroups “A & B” of G 2 “VITA ENAMIC” endocrowns (Table 3; Fig. 9). That finding which was incongruent with D’Arcangelo, et al [50], and agreed with Bindl and Mörmann [15, 55, 56], who confirmed remarked improvement in endocrown fracture resistance with the cavity depth and angle degree of occlusal reduction.

In addition, G1 ″NanoKsa G-PLUS″ consistently exhibited lower mean margin gap values (i.e. best marginal adaptation of all subgroups) compared to G 2 ″VITA ENAMIC″ across both preparation designs (Table 2; Fig. 8); which might be due to the inherent polymeric nature of the endocrown biomaterial, the 3D printing customizing the margin as well as the applied bonding system and adhesive resin cement materials that might resist the hydrolytic and/or oxidative degradation during the thermocycling procedure. That interpretation might be congruous with Wang, et al. [55].

Concerning the marginal gap, analysis of obtained subgroup results within each endocrown material group “G1 and G2; Nanoksa G -PLUS and VITA ENAMIC”, revealed acceptance of the 2nd suggested null hypothesis; as there was statistical difference in marginal gap distance mean values between the applied design preparation “Subgroup A and B; anatomic and butt joint” designs; (Table 2; Fig. 8). Those findings agreed with other study reports [42] that confirmed that type of endocrown biomaterial was significantly affecting the fracture resistance and the marginal gap distance of single-tooth restorations, however the implemented technology of material construction didn’t. Therefore, combined selection of the appropriate fabrication technique with the accurate recent endocrown biomaterial is considered a prerequisite for a successful durable restoration [19–21].

Marginal fractures of *“VITA ENAMIC” CAD/CAM* ceramic endocrown restorations might had occurred because of the exerted pressure from the milling machine instrument and fracture resistance of the brittle material crystallites that created micro-cracks and underwent crack propagation. That interpretation might be consistent with a previous related study [56]. However, the *3D* printing technology allowed an efficient flowability and compressibility of the high-performance polymers *“Nanoksa G-PLUS”* containing Nano zirconia and Nano carbon crystals [57]. That increased fracture resistance might be attributed to the crack deflection following crack propagation, which agreed with previous research study [42, 57]; where the authors referred the increase in viscosity of the studied biomaterial during heat pressing to the addition of zirconia nanocrystals and the subsequently decreased crystal growth without any marked increase in strength values.

Biomimetic technology of recently innovated and manufactured “Nanoksa G-Plus” high performance polymer and “VITA ENAMIC” hybrid ceramic are ceramic-polymer composites that endow the composite endocrowns’ microstructures with amazing mechanical properties simulating those of dentin and enamel. Polymer-infiltrated ceramic “PIC” as has been nominated for “VITA ENAMIC”, revealed better results than 3D printed high performance polymer “Nanoksa G-PLUS regarding the fracture resistance of the glass ceramics.

Declared limitations of that study included: The cement thickness and internal adaptation that influence stresses’ distribution and restorations’ longevity, were not evaluated, even though the adhesive bonding system used with each endocrown material and surface treatments in the study closely adhered to the manufacturers’ recommendations. Additionally, failure mode analysis was not performed and fractographic or micro-CT investigations are recommended for a thorough fit evaluation in future research.

## Conclusions

Within the scope of investigated parameters and limitations of the current study, the following conclusions can be addressed:

1. Smart technology of 3D printed Nanoksa G-PLUS high-performance polymers may offer a valued marginal adaptation for posterior endocrown restorations specially with butt joint preparation designs.
2. *CAD/CAM* VITA ENAMIC hybrid ceramic technology for posterior endocrown restorations specially with application of anatomic design preparations seems promising to improve fracture resistance of endocrown restorations in posterior teeth.
3. The effect of endocrown material inherent characteristics on marginal adaptation and fracture resistance are pretended consistent and may not significantly alter with the implemented molars ‘preparation designs “i.e. anatomic and butt joint” based on the specific preparation design used.

## Recommendations

### Clinical significance

Based on the evaluated in-vitro conditions, Nanoksa G-Plus and VITA ENAMIC hybrid ceramics appear to be promising endocrown restorations for posterior human permanent molars that can sustain high stresses. The clinical performance of these recently developed endocrown biomaterials will be emphasized by more clarification using finite element modeling and long-term clinical trials.

### Clinical relevance

The clinical performance of the adhesive systems in relation to the anatomic and butt joint design preparations employed for the investigated Nanoksa G-PLUS and VITA ENAMIC endocrown restoration may be clarified in future research, including the fractographic mode of failure, under the evaluated in-vitro settings.

## Data Availability

All data are provided in this study and raw data can be requested to corresponding author.

## Abbreviations

CAD/CAM: Computer Aided Design/Computer Aided Manufacture
3D: Three dimensional
EET: Endodontically treated teeth
G1: Group1, received 3D printed Nanoksa G-PLUS high-performance polymer endocrown restorations
G2: Group2, received CAD/CAM VITA ENAMIC hybrid ceramic endocrown restorations
G1A: 3D printed Nanoksa G-PLUS high-performance polymer endocrown restorations with a standardized anatomic preparation design
G2A: CAD/CAM VITA ENAMIC hybrid ceramic endocrown restorations with a standardized anatomic preparation design
G1B: 3D printed Nanoksa G-PLUS high-performance polymer endocrown restorations with a standardized butt joint preparation design
G2B: CAD/CAM VITA ENAMIC hybrid ceramic endocrown restorations with a standardized butt joint preparation design
n: Number of replicas in each group
ANOVA: Analysis of Variance
RFT: Root filled teeth
PEEK: Polyetheretherketone
ZrO_2_: Zirconia
STL file: STereoLithography; is a file format native to the stereolithography CAD software created by 3D Systems
CNC: Computerized Numerical Control
SPSS: Statistical Package for Social Sciences
M: Mean
SD: Standard Deviation
N: Newtons
Al_2_O_3_: Alumina or Aluminum Oxide
HF: Hydrofluoric Acid
secs: Seconds
min: Minutes
cm: Centimeter
µm: Micrometer
mm: Millimeter
°C: Degree Celsius
